# Mr. Thomson, on Hydrophobia

**Published:** 1808-11

**Authors:** Henry A. Thomson

**Affiliations:** Kensington


### *To the Editors of the Medical and Physical Journal.*

Gentlemen,

As there are still, I believe, many who disbelieve in the existence of Hydrophobia, I think it right that as much publicity as possible should be given to every case that may occur. By such multiplied evidence, these sceptics must be at length convinced, and may be saved from falling into a fatal security; and by a faithful report of every instance, the attention of able men may be drawn to the subject, and some new light probably thrown on a disease which has hitherto baffled the power of medicine.

Elizabeth Sharpe, aged 59, a small and delicate woman, was bitten in the hand on Sunday, July 24th, by a lap dog, which had followed her sister home that morning; and was detained with the hope of ascertaining its owner. In the course of the day, three children who were playing with it, were also bitten; and on the following morning the dog was driven from the house, and seen by them no more.

By some strange infatuation, no attention was paid to these circumstances, but the wounds (all of which were trifling) were left unheeded. In this person, the injury was inflicted over the first joint of the thumb of the right hand. The wound continued sore and troublesome for a few days, when a dry scab was formed over it, and the occurrence no longer noticed. The woman continued perfectly well until Sunday the 14th of August, three weeks from the time of the accident; when in chopping some wood, she run a small splinter into her thumb near the bitten part, from which there was a small oozing. She soon afterwards complained of much pain in that thumb and wrist, which increased in violence, and gradually extended up the outer side of the arm to the shoulder.

On Tuesday morning, August 16, she came to me, complaining of great pain in the right arm and shoulder, which, as she had been subject to the rheumatism, and none of the above-mentioned facts were disclosed, was treated as such. In the evening the pain had increased, and she declared herself unable to swallow liquids. I was called to her about 8 o'clock, and found her sitting up in bed, with a marked expression of anxiety in her countenance, though perfectly unsuspicious of the nature of her disease. She complained of great pain and oppression at the præcordia, with a sense of constriction in the throat. Her pulse was 120, small and hurried, and she was distressed by great thirst, with frequent frequent retchings. Her respiration was difficult and oppressed, and the pain from the arm had extended into her right breast. Having observed some rag rolled over her hand, I enquired into its use, and then discovered what has been above related; and on offering her some fluid in a tea-cup, had but too much reason to be convinced of the nature of her wretched disorder. Every attempt produced the most violent spasms in her throat, even before the vessel had reached her mouth.

It appeared, that during the greater part of the day she had found some difficulty in taking liquids; but which had very much increased towards evening.

℞. Opii purif. Ammon. pptt. a. gr. ij. Camphor. gr. iij. M. f. Pilul. 4tis horis sum. Applic. empl. canth. scrob. cordis. Applic. empl. ampl. ex confect. Opiat et camph. pectori. Infricet. brachiis ambob. ung. hydr. f. camphor. ʒij. et 6tis horis repet.

Fomentations were ordered also to be applied to her feet; and her arms to be washed with vinegar, previous to the application of the ointment. When the cold vinegar touched her skin, a sensation of great horror and uneasiness was produced, but which was not of long duration. In taking her second pill, she asked for some honey, and when she had helped herself, thrust away the bason as if distressing to her sight.

August 17th. I visited her this morning at 7 A. M. and found my patient had passed a sleepless and most restless night, though the pain and oppression at her stomach were in some degree relieved. She had swallowed, in the course of the night, three tea-spoonsfull of brandy and water, but not without the greatest difficulty, and violent spasms being each time produced. The ointment has been twice used, and the pills regularly taken.

℞. Persist. in usu pilul. cum addit. Calomel. gr. j. dosi singul. 4tis horis sumend. Persist. in usu unguent.

At 1 o'clock P. M. the difficulty of breathing was much increased, and she complained of a sense of suffocation from the wind rushing forcibly from the stomach into her throat whenever she attempted to swallow. No inflammation could be perceived in the course of the lymphatics, nor tenderness in the axillary glands. She had taken a few pieces of veal-cutlet into her mouth, chewed, but could not swallow them. She now ate with great resolution, one small portion of sopped bread, but it was evidently a considerable exertion; and after several efforts, succeeded in carrying a tea-spoonfull of liquid twice or three times to her mouth, mouth, swallowing a small quantity at each attempt; but when a cup was offered her, before it could approach her mouth, she was always violently convulsed.

℞. Opii purif. gr. j. Calomel. gr. ß. Camphor. gr. iij. M. f. pilul. quaq. hora sumend. Infric. femorib. et brachiis ung. hydrarg. fort. ʒij. 4tis horis.

10 o'clock, P. M. The patient considerably worse. The difficulty of swallowing and dyspnœa much increased; often gasping for breath from a sensation of suffocation, and her countenance expressing the utmost horror and anxiety. Her pulse 140, and very feeble. She had perspired freely, and in the forepart of the evening appeared in some degree composed; but having unfortunately overheard some conversation about the dog, incautiously held in the room, she became sensible, for the first time, of her truly wretched situation, and was deeply affected at the disovery. Since that time she has been much harrassed by convulsive fits, which occur very frequently, but at uncertain periods; and during which, supported by some attendants, she rushes forward a few paces, as if to relieve herself from some confinement; she is always aware of their approach, and prepares herself to meet them, by taking fast hold of two persons, assuring them at the same time, that they shall receive no injury. She is now extremely sensible of the least noise, and frequently speaks a few words to some ideal object; and then, recovering herself, inquires what she had seen.

Her eyes are turgid and appear thrust forward, and her other features are much distorted. She now finds great difficulty in swallowing the pills, and has succeeded in getting down only three. She attempted to suck a slice of cucumber, but every trial brought on the convulsive fits.

Sumat. tinct. opii, ʒß. 2da. quaq. hora. Injiciet. enema cnm tinct. opii, ʒiß. Applic. empl. canth. ampl. inter scapul. Persist. in usu unguent.

August 18, A. M. Found my patient had expired about half an hour, after a violent convulsive struggle, which lasted about five minutes, but ceased a few seconds before she breathed her last. She had suffered much through the night, from the repeated convulsions; during the intervals she rambled occasionally, but was perfectly sensible when roused, even to the last half hour; and appeared to take great pleasure in rubbing a table in the room, folding and putting away her clothes, &c. when the convulsions would permit.

Her

Her greatest concern was lest she should injure her sister; and to prevent that, strenuously requested that she might be confined in a room by herself. In the course of the night, her mouth became much affected by the mercury, the gums were swollen, and the secretion of saliva considerably increased.

She had also two copious evacuations of urine, which passed involuntarily and unconsciously during the convulsive paroxysms.

She had taken the laudanum only three times; and they had not been able to administer the injection.

Neither the opium, which was taken freely, nor the mercury, which produced its full effect on the system, appeared to have the least influence on this disease.

She had not a moment's sleep, from the first accession of the hydrophobic symptoms, to the time of her dissolution; a period of about forty-five hours.

From the situation of the family, it was not possible to examine the body.

---

Of the three children who were also bitten, one belonged to a friend, and was soon after removed to London; the others, (one about seven, the other fourteen years old) to the sister of the deceased, who kept the house.

The wounds which were on their hands were trifling, and in a few days perfectly cicatrized; nor was there in either the least appearance of disease. It was deemed expedient however, as the best means of prevention, that the parts should be destroyed, and the patients put under the influence of mercury; the former was effected with the calx cum kali puro; and after the separation of the sloughs, the wounds were dressed with a stimulating ointment, and discharged freely for three or four weeks. For the latter, the mercurial friction was employed, and a moderate affection of the mouth kept up for about a fortnight. The children have hitherto continued perfectly well; but whether their safety may be in any degree attributed to the treatment they have undergone, must remain for future experience to determine.

---

Having discovered the owner of the dog, a respectable gentleman in the neighbourhood, I applied to him for a particular account of all he had observed in the animal; and the following is a brief abstract of the statement with which he has obligingly furnished me.

In the last two years, the dog has been frequently unwell, and particularly after the three last litters, when all its its puppies were dead. In the beginning of July, it was observed to be very sickly; it ate little, and appeared heavy and dull. One of the children making a noise with a kettle on the pavement, the dog snarled and attempted to bite it. This being unusual, and reports of mad dogs prevalent in the neighbourhood, the family were alarmed, and offered the animal some water; she refused it at the moment, but afterwards drank freely, and their apprehensions were removed. About the middle of the month, she appeared to be perfectly recovered, eating heartily, and as playful as usual.

On Friday July 22d, she was observed to be at heat; and being found before the house surrounded by dogs, was driven in by the family. On Sunday morning the 24th, she was seen in the garden, eating currants, &c. from the bushes, and afterwards escaped from the premises. It then appears, that she went home with the sister of the deceased, and was detained by them till the following morning; while there, it refused all food, and was generally lying under the tables, &c.

On Monday the 25th, in the forenoon, the dog was seen passing the residence of its owner, and was called by some of the family; it turned immediately and went into the house. In the hall a child attempted to stroke it, the dog snarled and the child was removed. This circumstance creating an alarm, the animal was confined in an out-house and supplied with food and water. She appeared anxious to be released, and frequently made a sharp, though not loud howl; but whether from impatience of confinement or a sense of pain, as the gentleman supposed, must be doubtful. No saliva appeared at the mouth. On Wednesday morning the 27th, some of the food had disappeared, but not being always watched, it had not been seen to eat. On the evening of that day, however, it *was observed lapping the water*.

On Thursday the 28th, early in the morning, it was found lying in the back part of the building, its head resting between its paws on the ground; when called it raised its head and looked up, but made no further attempt to move; in half an hour it was again visited, and discovered stretched out on its side dead.

I am, &c.

HENRY A. THOMSON.

*Kensington, Oct.* 15, 1808.

